# Timeline representation of clinical data: usability and added value for pharmacovigilance

**DOI:** 10.1186/s12911-018-0667-x

**Published:** 2018-10-19

**Authors:** Thibault Ledieu, Guillaume Bouzillé, Frantz Thiessard, Karine Berquet, Pascal Van Hille, Eric Renault, Elisabeth Polard, Marc Cuggia

**Affiliations:** 1 INSERM, U1099, F-35000 Rennes, France; 2 Université de Rennes 1, LTSI, F-35000 Rennes, France; 3 CHU Rennes, F-35000 Rennes, France; 4 ERIAS, INSERM U897, ISPED, Université Bordeaux, Bordeaux, France; 5 Centre Régional de Pharmacovigilance, CHU Pontchaillou, Rennes, France

**Keywords:** Informatics, Information visualization, Pharmacovigilance, Usability testing

## Abstract

**Background:**

Pharmacovigilance consists in monitoring and preventing the occurrence of adverse drug reactions (ADR). This activity requires the collection and analysis of data from the patient record or any other sources to find clues of a causality link between the drug and the ADR. This can be time-consuming because often patient data are heterogeneous and scattered in several files. To facilitate this task, we developed a timeline prototype to gather and classify patient data according to their chronology. Here, we evaluated its usability and quantified its contribution to routine pharmacovigilance using real ADR cases.

**Methods:**

The timeline prototype was assessed using the biomedical data warehouse eHOP (from *entrepôt de données biomédicales de l’HOPital*) of the Rennes University Hospital Centre. First, the prototype usability was tested by six experts of the Regional Pharmacovigilance Centre of Rennes. Their experience was assessed with the MORAE software and a System and Usability Scale (SUS) questionnaire. Then, to quantify the timeline contribution to pharmacovigilance routine practice, three of them were asked to investigate possible ADR cases with the “Usual method” (analysis of electronic health record data with the DxCare software) or the “Timeline method”. The time to complete the task and the data quality in their reports (using the vigiGrade Completeness score) were recorded and compared between methods.

**Results:**

All participants completed their tasks. The usability could be considered almost excellent with an average SUS score of 82.5/100. The time to complete the assessment was comparable between methods (*P* = 0.38) as well as the average vigiGrade Completeness of the data collected with the two methods (*P* = 0.49).

**Conclusions:**

The results showed a good general level of usability for the timeline prototype. Conversely, no difference in terms of the time spent on each ADR case and data quality was found compared with the usual method. However, this absence of difference between the timeline and the usual tools that have been in use for several years suggests a potential use in pharmacovigilance especially because the testers asked to continue using the timeline after the evaluation.

**Electronic supplementary material:**

The online version of this article (10.1186/s12911-018-0667-x) contains supplementary material, which is available to authorized users.

## Background

Pharmacovigilance is defined by the World Health Organization (WHO) as “*the science and activities relating to the detection, assessment, understanding and prevention of adverse effects and any other drug-related problem.*” [1] Pharmacovigilance requires the retrospective collection of information from the patients’ medical records in order to find clues concerning drug accountability in the occurrence of adverse drug reactions (ADR). This means finding relevant information in patient records that may be evidence of an ADR. Clinical data are heterogeneous in terms of structure (structured or textual data) and domain (e.g., clinical/laboratory results or prescription data). Searching and organizing such information require specific expertise and is often a time-consuming task due to the lack of adapted tools for this purpose.

The re-use of clinical data from patients has been facilitated by the emergence of querying interfaces for clinical data warehouses, such as i2b2 tools [2] or complete systems (database and interface), such as eHOP [3]. These systems allow querying and analysing heterogeneous clinical data, and have been successfully exploited for clinical research (e.g., feasibility studies, patients’ pre-screening and recruitment for clinical trials [4]) and epidemiological studies (cohort detection) [5]. Pharmacovigilance is another field where the exploitation of health databases could play a key role [6].

Among the different data-mining methods, visual data analysis could be useful for exploring complex data. Indeed, visualization of data on the history of individual patients or groups of patients could allow highlighting chronological clues and causal relationships between drug exposure and ADR. A timeline is an interactive chronological representation of a list of events that can include different types of data and time scales. In the literature, several timeline tools for electronic health record (EHR) data visualization to explore individual or population-wide clinical data (e.g., Lifeline [7], Outflow [8], VisuExplore [9], Eventflow [10]) have been described. For instance, CareCruiser [11] provides simultaneously a view of the patient’s data and therapeutic protocols for the analysis of treatment responses (e.g., for a patient on oxygen, the O_2_ saturation values will vary depending on the treatment). VisuExplore [9] offers different types of graphs (distribution curves, histograms) depending on the data type (e.g., for a diabetic patient, insulin administration is displayed as dots, while biological parameters, such as glucose concentration, are displayed as distribution curves). KNAVE-II [12] is an interactive and semantic viewer of clinical data based on the use of domain ontologies. However, none of these tools has been evaluated for pharmacovigilance.

This work focused on a timeline tool developed in the framework of the Retrieval and Visualization of Electronic Health Records (RAVEL) [13] and Pharmaco-Epidemiology of Health Products (*Pharmaco-Epidémiologie des Produits de Santé,* PEPS) research projects the main mission of which is the development of new methods for information research and visualization of heterogeneous clinical data. In this paper we present the evaluation of the usability of this prototype timeline and the quantification of its contribution to pharmacovigilance routine practice in terms of time saving and quality of the collected data.

## Implementation

### Timeline description

**Fig. 1:**
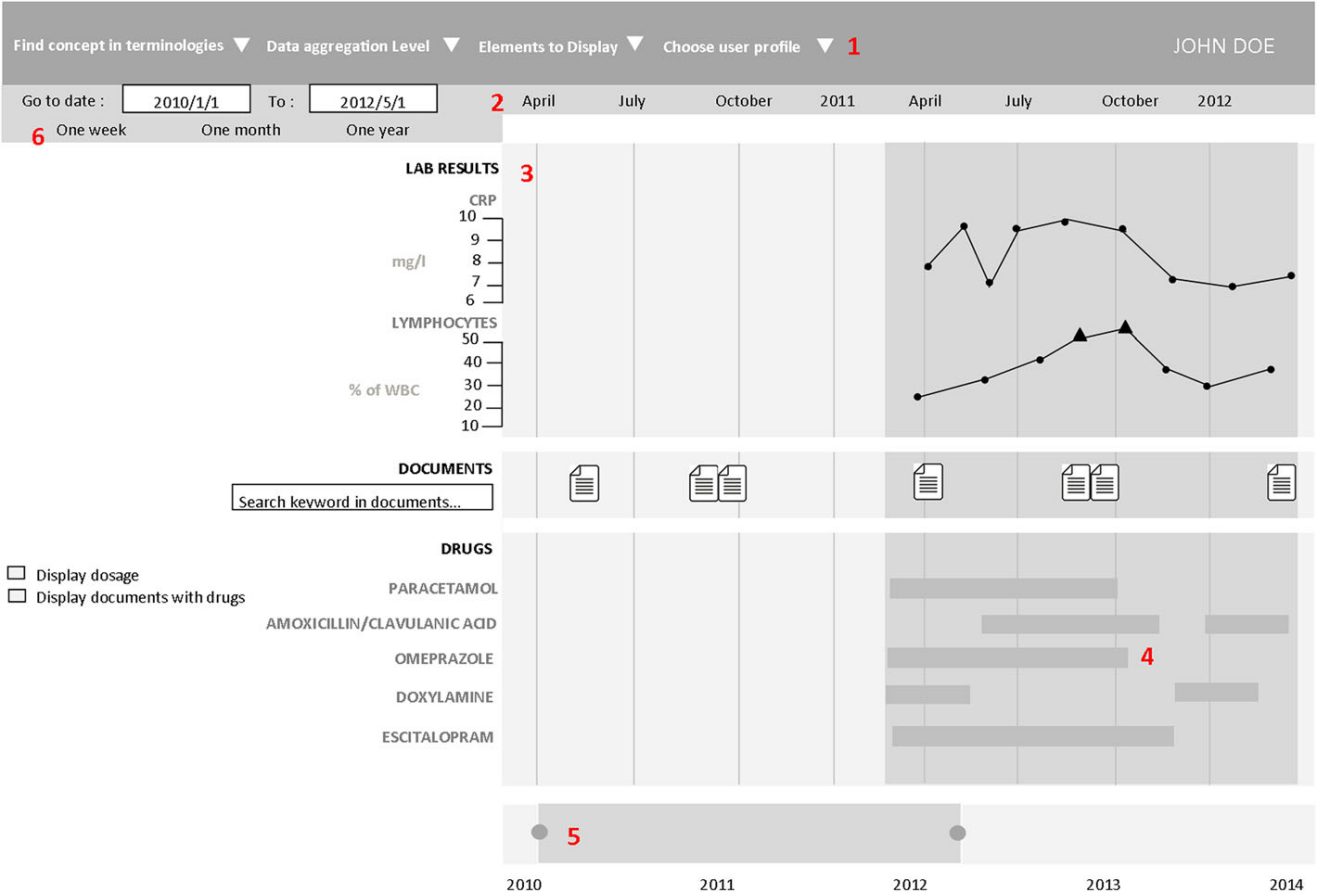
Timeline interface. The interface individual components are: 1. Selection of patient, laboratory and clinical data and medical codes (e.g., ICD-10 code). 2. Time scale. 3. Laboratory results in the form of graphic curves (dots show normal values; triangles and their orientation represent anomalous values). 4. Drug prescriptions and treatment duration (start, end and overall duration time, as well as the drug regimen. 5. Timeline overview for navigation. 6. Overall time period selection 4.

The prototype’s graphical user interface (GUI) was developed using the JavaScript d3.js library [14]. For a broad use, this component was designed as a web service, weakly coupled to the EHR data source (eHOP for the prototype). eHOP (from *entrepôt de données biomédicales de l’HOPital*), the biomedical data warehouse used by Rennes University Hospital Centre, contains comprehensive laboratory and clinical data on 1.6 million patients. It provides access to heterogeneous clinical information in the form of free text (hospitalization reports, discharge letters, etc.), of data coded using different terminologies (e.g., the International Classification of Diseases, 10th revision, ICD-10, medical diagnosis codes), and structured data (laboratory results, prescriptions and drug administrations). The prototype used a web interface to allow interactive navigation. Real patient data were used for the prototype assessment. Figure 1 is a screen mock-up in English to show how the information is displayed in the timeline. A real screenshot (in French) and demo are available in Additional file 1.

To the best of our knowledge, this application includes new features. Specifically, semantic data aggregation/expansion was implemented by leveraging the hierarchical properties of different terminologies (Anatomical Therapeutic Chemical, ATC, classification system; French Common Classification of Medical Procedures, CCAM; ICD-10; and the local terminology used by the Clinical Laboratory of Rennes). This approach allows users to interactively control the granularity of the displayed information.

Medical codes (e.g., ICD-10 codes) can be directly searched in different terminologies using a hierarchical tree. This tree also allows users to choose the structured data they wish to display in the timeline. The choice of concepts to display can be pre-set according to the user’s specific medical field. The user also can search for terms in text documents via a free text search field. Each type of data has its own representation mode: graphs for clinical test results, windows for textual records and iconic language for medical terminologies (Visualization of Concepts in Medicine icons) [15]. The user can expand or reduce the time scale at will through two navigation controls: the overall time period selection and the timeline overview.

All these features were designed to facilitate navigating through the complexity of patient data.

### Recruitment of experts

Expert users were recruited from a team of pharmacists and physicians who work at the Regional Pharmacovigilance Centre in Rennes and who are part of the target audience for our tool. Based on literature data [16], it was determined that five users were sufficient to identify 80% of the problems linked to the application’s usability [17]. Finally, six people (five women and one man among whom there were five pharmacists and one physician) could be recruited for the usability study, and three of them were also involved in the impact quantification study. These participants perform routinely pharmacovigilance activities. The six people received no financial compensation in exchange for their participation. Their mean age was 31.3 ± 4.5 years (range: 25 to 40). All routinely used computers as part of their pharmacovigilance activity, particularly to study patient records.

### Usability evaluation method

Usability is defined by the International Organization for Standardization (ISO) as the “extent to which a product can be used by specified users to achieve specified goals with effectiveness, efficiency and satisfaction in a specified context of use”. [18] There are many methods for evaluating usability [17] and several are used for the development and evaluation of health-related software programs [19–21]. We chose the ‘think-aloud protocol’ approach that has been described by Nielsen as “the most reliable evaluation method to obtain a usability estimate” [22]. It is based on recording the participants’ opinion while they perform a series of tasks designed specifically for the usability evaluation [23]. Participants are asked to orally state their thoughts, perceptions and opinions while interacting with the application (concurrent think aloud method). The participants’ performance during the execution of these tasks is then analysed by the evaluator. This method can be applied throughout all stages of the web application development and provides qualitative data that will help evaluators to identify usability problems. It can also prompt the development of new features for the interface in response to the users’ feedback. The severity of the usability problems encountered by users was rated with the Nielsen rating scale (from 0 = No usability problem to 4 = Usability catastrophe) [24]. Peute and al. [25] showed that compared with the retrospective think aloud method, the concurrent method increases participants’ verbalization and leads to a higher detection rate of usability problems. In addition, this method seems to be more reliable than the retrospective analysis of the test record, conducted in the users’ presence.

### Usability evaluation use case

An ADR case, chosen in collaboration with the head of the Pharmacovigilance Centre, was used to test all the prototype’s features in a real-life situation. Participants were asked to determine whether or not the adverse reaction experienced by a patient admitted in intensive care resulted from exposure to an antibiotic. To this aim, they had to perform four main tasks: i) to find the patient’s name and address, ii) to determine the exact nature of the adverse reaction and collect all pertinent information, iii) to find information on the suspected drug (name and dose, for instance) and iv) to collect information on the ADR outcome.

### Usability evaluation procedure

Before the usability test, all users underwent, at the same time, training to become familiar with the timeline functionalities for about one hour. For this training session, fictitious patient data were created in the same format as the actual data to be able to show/test the features of the timeline without having to access real patient data. During this training session, participants could visualize the data of a fictitious patient in the timeline (e.g., drug prescriptions, laboratory data, reports) and could use each functionality at least once (see Timeline description chapter for details on these functions). If a user encountered a problem, the trainer helped him/her to overcome this problem. This training session and the usability test of the prototype took place at the Medical Informatics Laboratory of Rennes University. The working environment (operating system, computers, etc.) was the same as the one used at the Pharmacovigilance Centre. Usability tests (six sessions with one participant per session) were performed in the presence of the evaluator, who observed the interactions between participant and interface and noted his/her observations on paper. Each participant received a notebook to record all relevant data retrieved on the use case. The usability testing sessions were recorded on video, as well as all interactions with the interface (keyboard input and mouse movements within the application). The recording and subsequent analysis were made using MORAE (TechSmith, software version 3.3.4). The video recordings were analysed after all participants had completed all the tasks. The MORAE software can capture on-screen video footage, while simultaneously recording the interactions between participant and GUI. Moreover, the participants’ face was also recorded, thus allowing the evaluators to assess, retrospectively, their expression and reactions while interacting with the prototype.

At the end of the usability test, each participant filled in the System and Usability Scale (SUS), translated into French. This questionnaire allows measuring the application’s usability in a fast and reliable way [26], and scoring was done as proposed by Sauro and Lewis. [27] Finally, each participant was interviewed using open-ended questions to determine the timeline features they most appreciated and whether they encountered any particular problems. In addition, users were asked about suggestions to improve the timeline. The interviews were not recorded.

### Quantitative impact study

For the impact study, the timeline prototype was slightly modified to take into account the suggestions/remarks from the six participants after the usability test (the added features are described in the Results section).

### Selection of patient records for the impact study

**Fig. 2:**
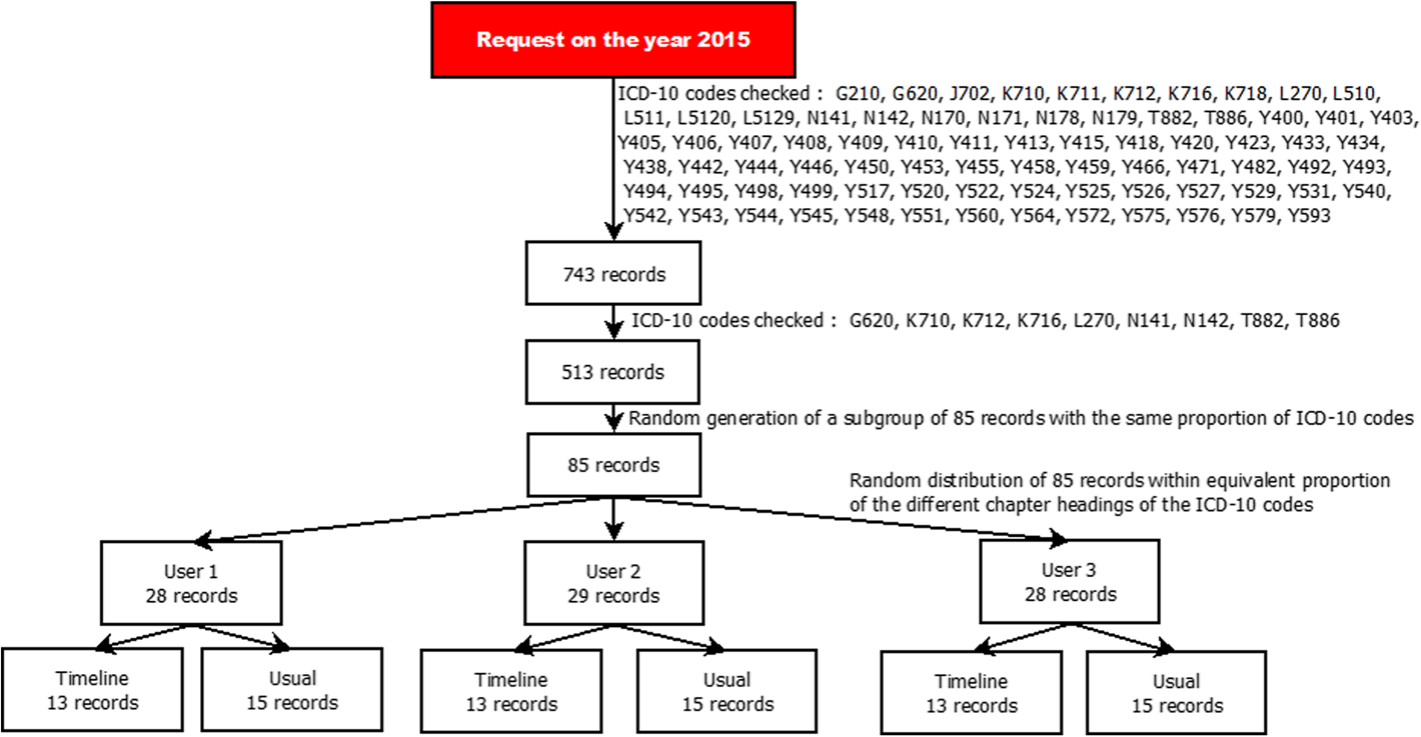
Randomization of patient records 8

For the quantitative impact study, 743 potential cases from the 2015 eHOP data were selected according to a list of ICD-10 codes that were potentially related to ADR and the method described by Osmont et al. [28]. This list was defined in a previous internal study of the Pharmacovigilance Centre based on 2014 data. Only ICD-10 codes with the highest probability of matching an ADR were selected (Fig. 2). Then, 85 cases were randomly chosen, in order to have roughly the same proportion of the different ICD-10 codes for the three participants and for each analysis method (usual method versus timeline, see below). A Python script was used to randomize the cases among the three participants.

### Procedure for impact quantification

These 85 cases were reviewed by three members of the Regional Pharmacovigilance Centre of Rennes using two methods: the “usual method” (EHRs and DxCare® software, see Additional files 2 and 3), and the “timeline” method (timeline prototype). Participants were provided with a list of patient records and instructions on which method to use for each record (Fig. 2). The working environment (operating system, computers, etc.) was the same as the one used at the Pharmacovigilance Centre. As in the routine pharmacovigilance practice, they had to gather information to establish a possible causality link between the diagnosis and the administration of a drug. They also wrote down the start time and end time for processing each file. The process was considered finished when the participant had retrieved all the required information or when he/she considered that this information was not present or could not be found with the used method.

After reviewing a case, participants filled in a home-made questionnaire detailing the following information: complexity of the case, information about the ADR, which functions of the timeline were used, and general comments.

The report quality was measured using a method based on the WHO vigiGrade Completeness score that includes quality indicators, such as the drug name or description of the ADR (see Additional file 4). Furthermore, absence of information affected the resulting score. A member of the Pharmacovigilance Centre not involved in the timeline testing did the scoring.

All analyses were conducted on the 85 cases, but for the vigiGrade Completeness score comparison that concerned only the cases considered to be ADR by the participants. Time differences between methods were also compared according to the participants and according to the chapter of the ICD-10 codes.

### Statistical analyses

Quantitative variables were expressed as mean and standard deviation. Continuous variables were compared using the Student’s *t* test or Wilcoxon rank-sum test, as appropriate, for two groups, and with the Kruskal-Wallis test for more than two groups. *P* values < 0.05 were considered significant. Analyses were performed with the R statistical software, version 3.2.2. [29]

## Results

### Usability evaluation

The participants’ average SUS score was 82.5 out of 100 points. After responding to the questionnaire, all participants expressed the desire to use the timeline in their daily practice.

All participants could successfully perform the tasks required for the use case. The use case completion time varied greatly among participants (mean completion time = 24.44 ± 9.97 min). The click count also was variable among participants (mean number of clicks: 174 ± 92.41, *P* = 0.06).

The error distribution in handling the interface did not vary much among participants (mean number of errors: 1.33 ± 1.5). The task to gather information on the ADR outcome had the highest error count (*P* = 0.16).

Table 1 lists the usability issues encountered by participants during the test. The severity of each usability problem was evaluated with the Nielsen rating scale [24] .

**Table 1:** Usability issues

Problem	Source of the problem	Problem frequency	Nielsen scale score	Observations
Zoom increase/decrease functionality deemed insufficiently accurate by participants	User Interface (UI)	6/6 (100%)	3/4	The Timeline overview does not allow accurate zooming to select the detailed view range
Absence of prescription data	Data source	6/6 (100%)	3/4	Prescriptions were not digitized in the Intensive Care Unit
Text highlighting did not work during the search	UI	1/6 (17%)	3/4	Issues with accent detection
Medication regimens were not displayed	UI	6/6 (100%)	3/4	Data on medication regimens were not available
Hierarchical tree of biological concepts: navigation problems	Complexity of the terminology in the UI	3/6 (50%)	2/4	The hierarchical tree of laboratory results was too large to allow users to easily find information without filtering
Repositioning document windows	UI bug	2/6 (34%)	2/4	The window slid under the menu bar

### Corrections / adjustments of the application

The usability assessment allowed users to make recommendations for improving the user interface. These recommendations have led us to adding new features designed specifically for pharmacovigilance, such as the option to automatically extract the drug names from textual documents and the ability to access, directly from the timeline, a module for visualizing the drug monography.

### Evaluation of the application quantitative impact on the routine practice

Three participants (two pharmacists and one physician) were recruited for this evaluation. All were also involved in the usability study.

**Fig. 3:**
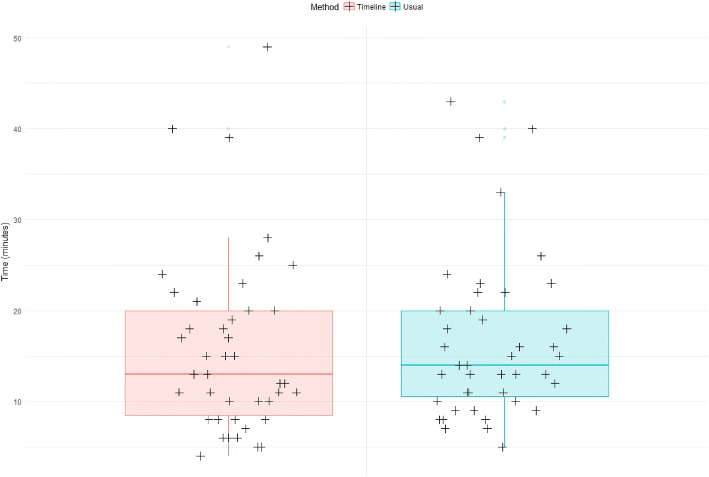
Box-plot displaying the time spent per case depending on the method 11

Overall, the time required for task completion was comparable between methods (16′ 29″ and 15′ 51″ for the usual method and the timeline, respectively; *P* = 0.38) (Fig. 3).

**Fig. 4:**
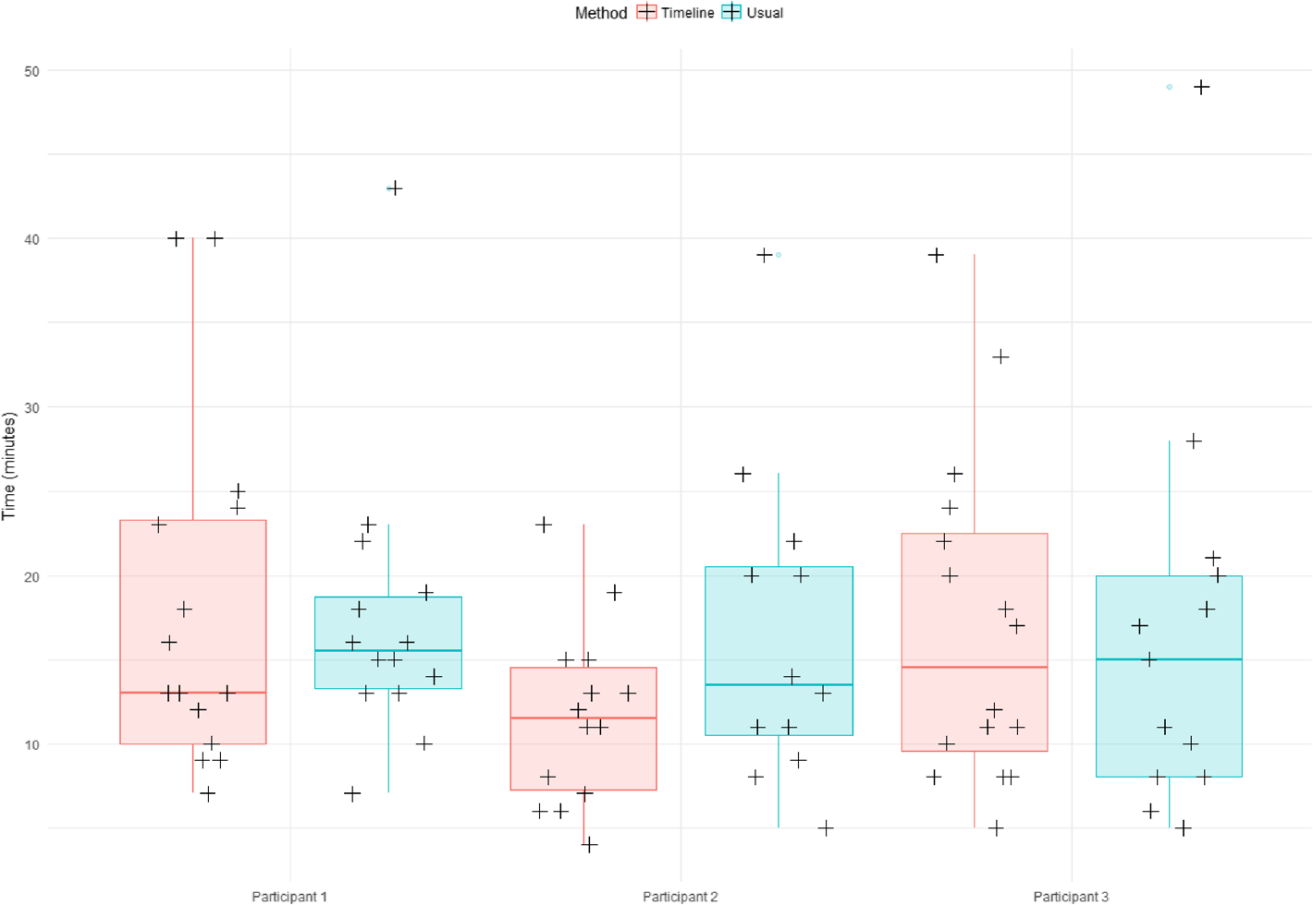
Box-plot displaying the time spent per case in function of the method and participant 12

Comparison among the three participants, showed that for user 1, completion time was significantly lower with the timeline than with the usual method (*P* = 0.02). No difference between methods was observed for the other two participants (Table 2 and Fig. 4).

**Table 2:** Average time spent per case in function of the method and the participant

Method Participant	Usual	Timeline	Difference	*P*-value
Participant 1	17′ 26″	11′ 38″	- 5′ 48″	0.02
Participant 2	15′ 55″	18′ 11″	+ 2′ 16″	0.71
Participant 3	16′ 8″	17′ 40″	+ 1′ 32″	0.59

Finally, the completion time was compared between methods and the type of ICD-10 chapter (Table 3). Although not significantly different, completion time tended to slightly faster with the timeline method than the usual method only for cases concerning genitourinary system diseases (*P* = 0.08). This difference could be explained by the systematic need to consult the patient’s laboratory results to judge the drug involvement in this type of ADR.

**Table 3:** Average time spent per case in function of the method and ICD-10 chapter

ICD-10 Chapter	Usual (n)	Timeline (n)	Difference
XIV - Diseases of the genitourinary system	17′ 36″ [20]	13′ 10″ [18]	- 4′ 26″
XII - Diseases of the skin and subcutaneous tissue	15′ 50″ [12]	15′ 20″ [9]	- 30″
XX - External causes of morbidity and mortality	15′ 17″ [7]	15′ 15″ [4]	- 2″
VI - Diseases of the nervous system	17′ [5]	20′ [9]	+ 3′
XI - Diseases of the digestive system	13′	17′ 45″	+ 4′ 45″
XIX - Injury, poisoning and certain other consequences connected with external causes	12′ 40″	14′ 12″	+ 1′ 32″

Among the 85 selected cases, 68 corresponded to actual ADR. Therefore, data quality was assessed and compared only for these records using the vigiGrade Completeness score. Overall, the average quality of the notifications (report for each adverse event) produced with the two methods was not significantly different (*P* = 0.49). Similar results were obtained also when comparing the notification quality among the three participants (Table 4).

**Table 4:** Mean notification quality according to the method and the user

Participant	Average quality, usual method	Average quality, timeline	Overall average quality
Participant no. 1	0.87	0.81	0.84
Participant no. 2	0.81	0.85	0.84
Participant no. 3	0.81	0.74	0.78
Total	0.83	0.80	0.81

### Participants’ feedback

During the interviews participants expressed the wish to use the timeline in their routine practice. The most appreciated features were the search functionality in the text and prescription display. The interviews also highlighted some negative points: problems in choosing the range of time of the timeline and difficulties in selecting the elements to display in the timeline. Moreover, the participants wished to see the drug regimens in addition to the prescriptions.

After the evaluation of the timeline impact on the pharmacovigilance practice, participants reiterated their interest in obtaining the application for use in their daily practice. They stated that the timeline allowed them to save time in all cases that required consulting laboratory results. Moreover, they added that the possibility to display data prescription and information on drug administration in a chronological format was a considerable advantage.

## Discussion

### Usability evaluation

The results of the timeline usability test were almost excellent, as indicated by the SUS score (82.5) and users’ feedback. The SUS questionnaire score indicates a good usability level. This score places the timeline in the 10% of applications with usability scores higher than 80%, [30] and is higher than that of the KNAVE-II tool (69.1) [31]. The reported usability problems were, in general, minor defects that could be rapidly corrected. Moreover, based on the users’ feedback, new functionalities were introduced to better meet the needs of pharmacovigilance users. The significantly higher number of errors during the task of searching the ADR outcome can be explained by the high complexity of this task (search involving laboratory results, reading several documents simultaneously, etc.). Differences in completion times can be explained by differences in each participant’s working method. Some participants carried out their tasks in parallel, while others performed them one after the other. This explanation also applies to the difference in the number of clicks, because some participants had to search for the same information more than once.

### Evaluation of the application quantitative impact on the routine practice

The evaluation of the timeline quantitative impact on the routine practice showed a significant gain of time for one of the participants. This user was the one who participated the most in the development of the application and had the least experience with the usual method, compared with the others. This observation suggests that the gain of time could have been more general with a longer preliminary training session to better familiarize all participants with the timeline functionalities. Moreover, as the timeline was compared to the method that has been used in the Pharmacovigilance Centre for more than 10 years, the overall absence of time-saving supports the need for a new evaluation after participants have become more accustomed to the timeline tool, especially because the users wanted to be able to continue using the timeline after the evaluation.

Overall, for simple cases for which retrieving the record of the hospital stay is sufficient, the timeline added value is less apparent.

Finally, the absence of any significant difference in the quality of reporting shows that the exclusive use of the timeline is not a limiting factor for data collection. Moreover, for some cases processed using the timeline, the poor quality of reporting could be explained by the presence of only textual documents in the application. This lack of data was not related to the timeline itself, but to the few data available in the data warehouse.

### Future versions

On the basis of the results and feedback obtained in this study, we are planning to introduce new features in the next version of the application. Particularly, we will focus on better displaying textual records, which is the starting point for locating information within patient records. Also, it will be possible to more easily compare the contents of a document with the structured data displayed in the timeline.

### Limits of the study

During the usability test, breaking down the use case into tasks caused issues in the analysis of the MORAE records. Indeed, these tasks did not occur sequentially, but in parallel because in pharmacovigilance the investigation process does not follow a single critical path. In addition, for the same task, participants did not search the same level of details. Some participants wanted very specific and detailed information (e.g., the half-life of the molecule administered to the patient), and therefore they required more time to complete their tasks. Conversely, other participants simply used the information contained in the text document retrieved by the query. It is, therefore, difficult to attribute the time differences observed during the execution of different tasks to an application usability problem.

For the impact study, only three participants were available, due to the limited human and material resources at our disposal, and therefore, it was not possible to process the same record twice using the two methods. A more thorough comparison between methods would require greater test power.

## Conclusion

The complexity and richness of data found in medical records require the development of tools for their efficient exploitation. Among the required features, viewing and querying information within a file remain critical elements. As part of the RAVEL project, we developed an integrated method for searching and visualizing information, in a timeline format. This article details the evaluation of this timeline. The results of the usability test indicate a good general level of usability, and new features adapted to pharmacovigilance routine practice could be added on the participants’ request. The timeline is now available to people in charge of pharmacovigilance for their daily practice.

## Availability and requirements

Project name: RAVEL Timeline.

Project home page: e.g. http://devtools.univ-rennes1.fr/websvn/Timeline/

Operating system(s): Platform independent.

Programming language: Java 8, HTML 5, CSS 3, JavaScript.

Other requirements: Java 8 or higher, Oracle 11 g or higher.

License: GNU GPL.

Any restrictions to use by non-academics: For use by non-academics, please contact Pr Marc Cuggia: marc.cuggia@univ-rennes1.fr

## Additional files

Additional file 1: Timeline interface (original French version). The interface individual components are: 1. Selection of patient, laboratory and clinical data and medical codes (e.g., ICD-10 code). 2. Time scale. 3. Laboratory results in the form of graphic curves (green dots are normal values, triangles and their orientation correspond to anomalous values). 4. Drug prescriptions and treatment duration (start, end and overall duration) as well as the drug regimen. 5. Timeline overview for navigation. 6. Overall time period selection. (PNG 140 kb)

Additional file 2: Portfolio interface (in French) (PNG 392 kb)

Additional file 3: Portfolio mock-up (PNG 63 kb)

Additional file 4: Dimensions included in the vigiGrade Completeness score and the corresponding penalties. (PNG 153 kb)

## Abbreviations

ADR: Adverse drug reaction
ATC: Anatomical therapeutic chemical terminology
CCAM: French classification, for the medical diagnostic or therapeutic procedures (*Classification Commune des actes médicaux*)
eHOP: Biomedical Data Warehouse of Hospital (*Entrepôt de données biomédicales de l’HOPital*)
EHR: Electronic health record
ETL: Extract transform load
GUI: Graphical user interface
HMI: Human-machine interface
ICD-10: International classification of diseases, 10th edition
PEPS: Pharmaco-epidemiology health products (*Pharmaco-Epidémiologie des Produits de Santé)*
RAVEL: Retrieval and visualization in electronic health records
SUS: System usability scale
UI: User interface
VCM: Visualization of medical knowledge (*Visualisation des Connaissances Médicales*)
WHO: World Health Organization
