# Supplementary figures and images for: Timeline representation of clinical data: usability and added value for pharmacovigilance
Source: BMC Med Inform Decis Mak. 2018 Oct 19;18:86. doi: 10.1186/s12911-018-0667-x (PMC6194681; doi:10.1186/s12911-018-0667-x)

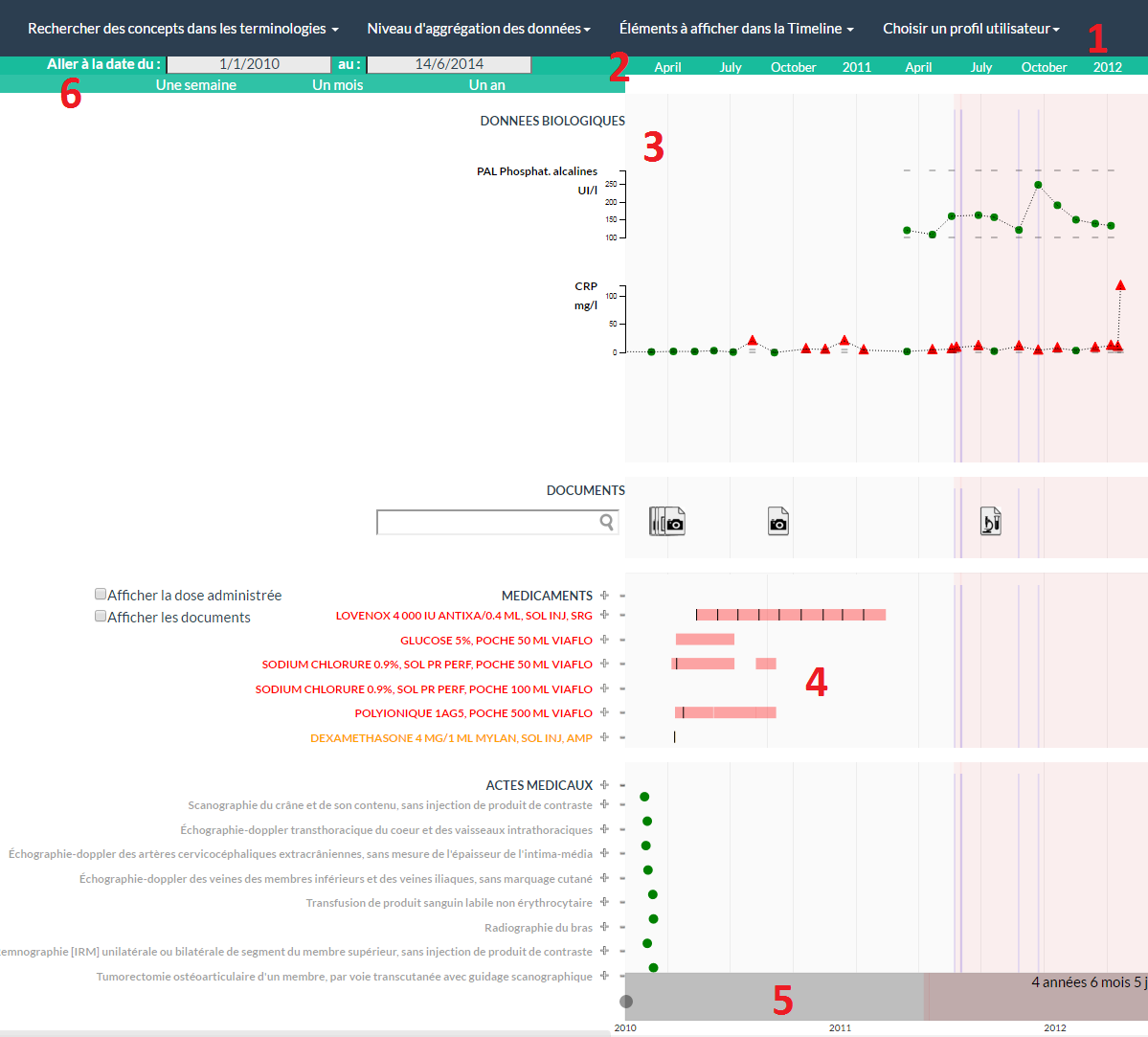

Supplement: Supplementary file 1 — Timeline interface (original French version). The interface individual components are: 1. Selection of patient, laboratory and clinical data and medical codes (e.g., ICD-10 code). 2. Time scale. 3. Laboratory results in the form of graphic curves (green dots are normal values, triangles and their orientation correspond to anomalous values). 4. Drug prescriptions and treatment duration (start, end and overall duration) as well as the drug regimen. 5. Timeline overview for navigation. 6. Overall time period selection. (PNG 140 kb) [file 12911_2018_667_MOESM1_ESM.png]

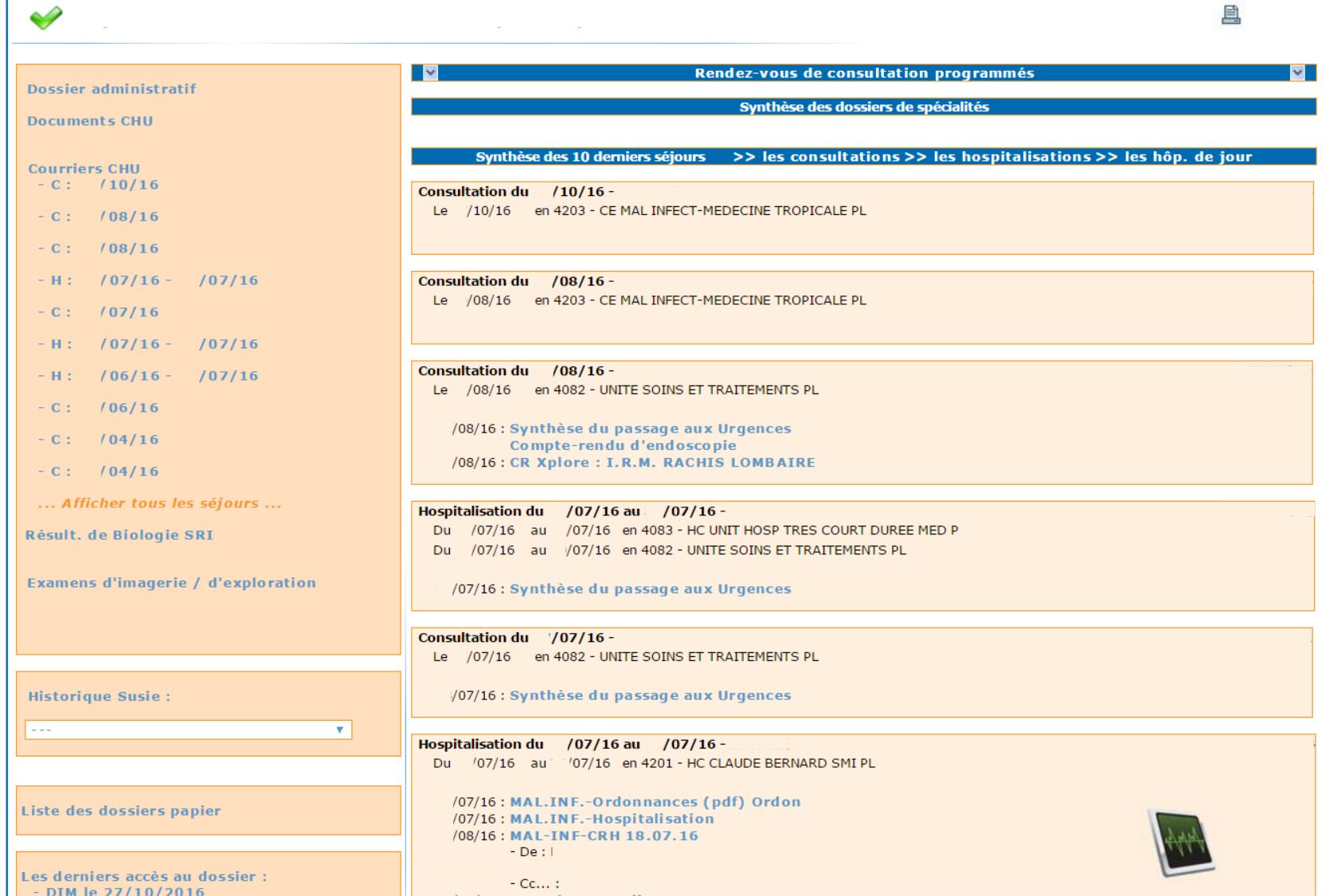

Supplement: Supplementary file 2 — Portfolio interface (in French) (PNG 392 kb) [file 12911_2018_667_MOESM2_ESM.png]

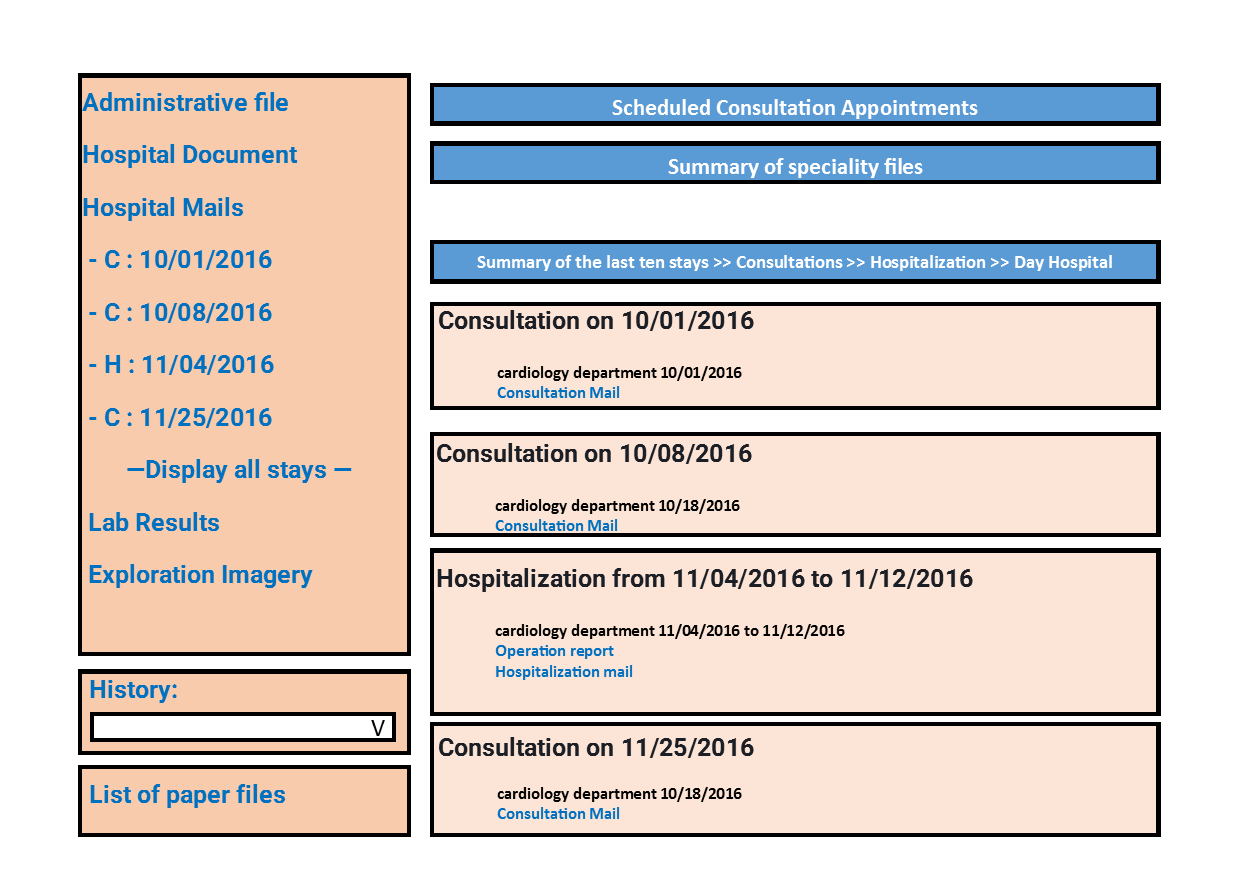

Supplement: Supplementary file 3 — Portfolio mock-up (PNG 63 kb) [file 12911_2018_667_MOESM3_ESM.png]

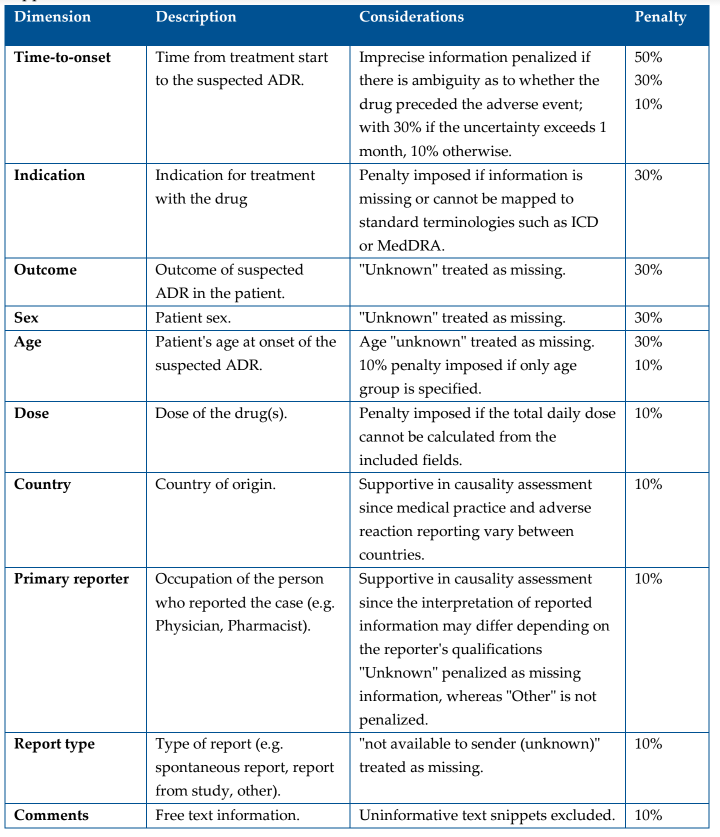

Supplement: Supplementary file 4 — Dimensions included in the vigiGrade Completeness score and the corresponding penalties. (PNG 153 kb) [file 12911_2018_667_MOESM4_ESM.png]
